# AxoNet: A deep learning-based tool to count retinal ganglion cell axons

**DOI:** 10.1038/s41598-020-64898-1

**Published:** 2020-05-15

**Authors:** Matthew D. Ritch, Bailey G. Hannon, A. Thomas Read, Andrew J. Feola, Grant A. Cull, Juan Reynaud, John C. Morrison, Claude F. Burgoyne, Machelle T. Pardue, C. Ross Ethier

**Affiliations:** 1 Wallace H. Coulter Department of Biomedical Engineering, Georgia Institute of Technology and Emory University, Atlanta, Georgia United States; 2 George W. Woodruff School of Mechanical Engineering, Georgia Institute of Technology, Atlanta, Georgia United States; 3 Center for Visual and Neurocognitive Rehabilitation, Atlanta VA Healthcare System, Atlanta, Georgia United States; 4 Devers Eye Institute, Legacy Research Institute, Portland, Oregon United States; 5 Casey Eye Institute, Oregon Health & Science University, Portland, Oregon United States

**Keywords:** Software, Image processing

## Abstract

In this work, we develop a robust, extensible tool to automatically and accurately count retinal ganglion cell axons in optic nerve (ON) tissue images from various animal models of glaucoma. We adapted deep learning to regress pixelwise axon count density estimates, which were then integrated over the image area to determine axon counts. The tool, termed AxoNet, was trained and evaluated using a dataset containing images of ON regions randomly selected from whole cross sections of both control and damaged rat ONs and manually annotated for axon count and location. This rat-trained network was then applied to a separate dataset of non-human primate (NHP) ON images. AxoNet was compared to two existing automated axon counting tools, AxonMaster and AxonJ, using both datasets. AxoNet outperformed the existing tools on both the rat and NHP ON datasets as judged by mean absolute error, R^2^ values when regressing automated vs. manual counts, and Bland-Altman analysis. AxoNet does not rely on hand-crafted image features for axon recognition and is robust to variations in the extent of ON tissue damage, image quality, and species of mammal. Therefore, AxoNet is not species-specific and can be extended to quantify additional ON characteristics in glaucoma and potentially other neurodegenerative diseases.

## Introduction

Glaucoma is the leading cause of irreversible blindness worldwide^1,2^, and thus is a significant research focus. This optic neuropathy is characterized by degeneration and loss of retinal ganglion cells (RGCs), which carry visual signals from the retina to the brain. Therefore, an important outcome measure in studying glaucomatous optic neuropathy, particularly in animal models of the disease, is the number and appearance of RGC axons comprising the optic nerve^3,4^, usually evaluated from images of optic nerve cross sections. Using images obtained by light microscopy is known to result in an axon count underestimation of around 30% relative to counts from images obtained by transmission electron microscopy^5,6^. However, light microscopy is widely used to count optic nerve axons because of its lower cost and favorable time requirements for tissue preparation. Therefore, in this work we focus on axon counting in optic nerve images generated by light microscopy.

Manual counting is the gold standard approach to quantify RGC axons, but it is extremely labor-intensive, since RGC axon numbers in healthy nerves range from the tens of thousands in mice to more than a million in humans^7^. Further complicating axon quantification is the fact that axon appearance can be highly variable. For example, in the healthy nerve, most axons are characterized by a clear central axoplasmic core and a darker myelin sheath; following previous work^5,8^, we will refer to such an appearance as “normal”. However, in damaged nerves (and even occasionally in ostensibly heathy nerves), other axon appearances occur, such as an incomplete myelin sheath and/or a darker axoplasmic region. Such variability further increases the time needed for axon counting, since the person doing the counting often needs to decide whether a given feature is (or is not) an axon. Here and throughout we place the term “normal” in quotes. An “abnormal” axon appearance does not necessarily imply non-functionality, and it is important to keep this distinction in mind.

To reduce the time-intensive counting process, various techniques have been developed for assessing axon counts and/or optic nerve damage, including: semi-quantitative, sub-sampling, semi-automated counting, and automated counting. In the semi-quantitative approach, scores based on a damage grading scale are assigned to optic nerves by different trained observers, and then averaged^8,9^. While this method is capable of quickly capturing whole-nerve changes, and can identify subtle changes that may not be detectable by axon counting, it is subjective and requires scorers who have significant experience and training. Sub-sampling is the process of estimating axon loss by manually counting smaller regions of the nerve using either targeted or random sampling and then extrapolating to the whole nerve or providing an RGC axon count per area measurement^5^. Sub-sampling is faster than full manual counting, but it is still labor-intensive and can be poorly suited to analyzing nerves with regional patterns of axonal loss^9^. Koschade *et al*. have recently presented an elegant stereological sub-sampling method that eliminates the bias that can occur in sub-sampling, but still requires manual axon counting in 5–10% of the full nerve area^10^. While this is feasible in animals with fewer axons per optic nerve like the mouse, counting this proportion may be prohibitive for animals with more axons per optic nerve, as in primates. Semi-automated axon counting methods use algorithmic axon segmentation techniques involving hyperparameters, such as intensity thresholds which are manually tuned for individual sub-images^11^. These methods are faster than manual counting and more thorough than qualitative or sub-sampling methods, but still require extensive human direction and time. Because of these limitations, there has been a push to develop fully automated counting tools.

Two of the most used automated counting tools are AxonMaster^12^ and AxonJ^13^. Both tools are designed to count “normal”-appearing axons, i.e. axons with a clear cytoplasmic core and a dark myelin sheath^5,8^. They use dynamic thresholding techniques to segment axonal interiors from myelin and other optic nerve features. While these tools are faster and provide more detail than sub-sampling methods, they also suffer limitations. For example, they are not easily extensible to counting features other than “normal”-appearing axons. Further, the two automated counting packages that currently exist were each developed for a specific animal species, and due to inter-species differences, it is not clear how accurate these approaches are for other species. Specifically, AxonMaster^12^ and AxonJ^13^ were calibrated and validated for use in non-human primate (NHP) and mouse models of glaucoma, respectively. Recently, AxonMaster has been applied to count RGCs in healthy and damaged tree shrew optic nerves^14^, but it has yet to be validated in this animal model. Our preliminary testing using these packages suggested that they are also sensitive to image quality, tissue staining intensity, and nerve damage extent in images of rat optic nerves (see Results).

Our goal was thus to create axon-counting software to overcome the above limitations, i.e. software which was robust to image quality and staining intensity, which could be used in multiple animal models of glaucoma, and which was extensible to quantification of features other than “normal”-appearing axons. Our approach to building this software, which we refer to as AxoNet, is an adaptation of the U-Net convolutional neural network architecture developed by Ronnenberger *et al*.^15^ applied to the count density learning approach of Lempitsky *et al*.^16^.

We used a dataset of manually annotated rat optic nerve images for developing and training AxoNet (detailed in Annotated Dataset Construction). The rat is a widely used animal model for glaucoma research and elevation of IOP produces retinal structural changes and loss of RGC axons similar to those observed in the human pathology^17^. We then applied our software to the dataset of NHP optic nerve images which was used to validate AxonMaster by Reynaud *et al*.^12^. Below we present the detailed methodology of the dataset and software construction used to develop AxoNet, as well as a comparison of AxoNet’s automated counting results to those of AxonMaster and AxonJ. We have packaged AxoNet into a user-friendly open source plugin for the widely-used ImageJ image processing platform^18^, as described in greater detail in the Discussion.

## Methods

### Rat optic nerve dataset

#### Animals

This study used twenty-seven optic nerves from fourteen (12 male and 2 female) Brown Norway rats aged 3 to 13 months (Charles River Laboratories, Inc., Wilmington, MA). All procedures were approved by the Institutional Animal Care and Use Committee at the Atlanta Veterans Affairs Medical Center and Georgia Institute of Technology and conformed to the Animal Research: Reporting of *In Vivo* Experiments (ARRIVE) guidelines. All experiments were performed in accordance with relevant guidelines and regulations. Rats used in this study had various degrees of optic nerve health. Each animal had one eye with experimental glaucoma induced unilaterally by either microbead injection (12 animals)^19–21^ or hypertonic saline injection (2 animals)^22^. Optic nerves in the resulting dataset ranged from ostensibly normal to severely damaged due to ocular hypertension. These 14 rats had been used in other studies and both optic nerves were used from each animal. One optic nerve was excluded from the study because it had suffered extreme damage secondary to abnormally high IOP elevation, which made it unsuitable for use in studying experimental models of chronic glaucoma.

#### Tissue processing and imaging

Animals were euthanized via CO_2_ and the eyes were enucleated. The optic nerves were transected with micro scissors close (<1 mm) to the posterior scleral surface. Optic nerves were then placed in Karnovsky’s fixative, post-fixed in osmium tetroxide, dehydrated in an ethanol series, infiltrated and embedded in Araldite 502/Embed 812 resin (EMS, Hatfield, PA). Semithin sections of 0.5 µm thickness were cut on a Leica UC7 Ultramicrotome (Leica Microsystems, Buffalo Grove, IL) and stained with 1% toluidine blue (Sigma-Aldrich, St. Louis, MO). They were imaged with a Leica DM6 B microscope (Leica Microsystems, Buffalo Grove, IL) using a 63x lens and 1.6x multiplier for a total magnification of 100×. A z-stack tile scan of the entire nerve was taken and the optimally focused image within each z-stack tile was selected using the “find best focus” feature in the LAS-X software (Leica Microsystems, Buffalo Grove, IL). Contrast was then adjusted for each tile by maximizing grey-value variance.

#### Annotated dataset construction

To train the AxoNet algorithm, it was necessary to create a dataset of rat optic nerve images in which axons had been identified. For this purpose, 12 × 12 µm sub-images were randomly selected from the full 27 nerves, producing a dataset of 1514 partial optic nerve images, with a minimum of 20 sub-images selected from each nerve, as follows:

- 200 images were taken from each nerve from the two female rats. These images were initially 48 ×48 µm, but were subdivided into 16 images, so that each sub-image matched the 12 × 12 µm standard image size.
- 50 images were taken from each nerve from an early cohort of four microbead model rats. The images from this source were initially 24 × 24 µm, and were similarly subdivided to yield 12 × 12 µm standard sub-images.
- 20 to 50 images, each of which was 12 × 12 µm, were selected from each nerve from a later cohort of eight microbead model rats.

All sub-images were 187 ×187 pixels, i.e. image resolution was 15.7 pixels per μm. However, during training and processing, the U-net architecture’s four max pooling layers each reduced the image side lengths by half, so all images used by AxoNet were required to have dimensions evenly divisible by 2^4^. To comply with this restriction, we used bilinear pixelwise interpolation to resize all dataset images to 192 × 192 pixels, i.e. to the dimensions closest to the images’ original size which were divisible by 16.

**Figure 1:**
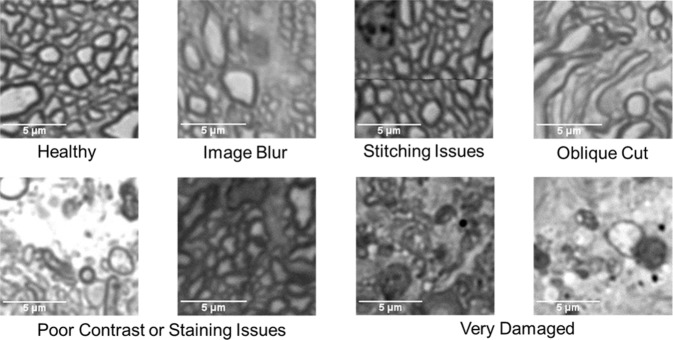
Rat Dataset Image Variety. A representative set of images from the rat optic nerve image dataset is shown. These images include a range of nerve health, variations in sample processing quality, and in image acquisition contrast and quality.

**Figure 2:**
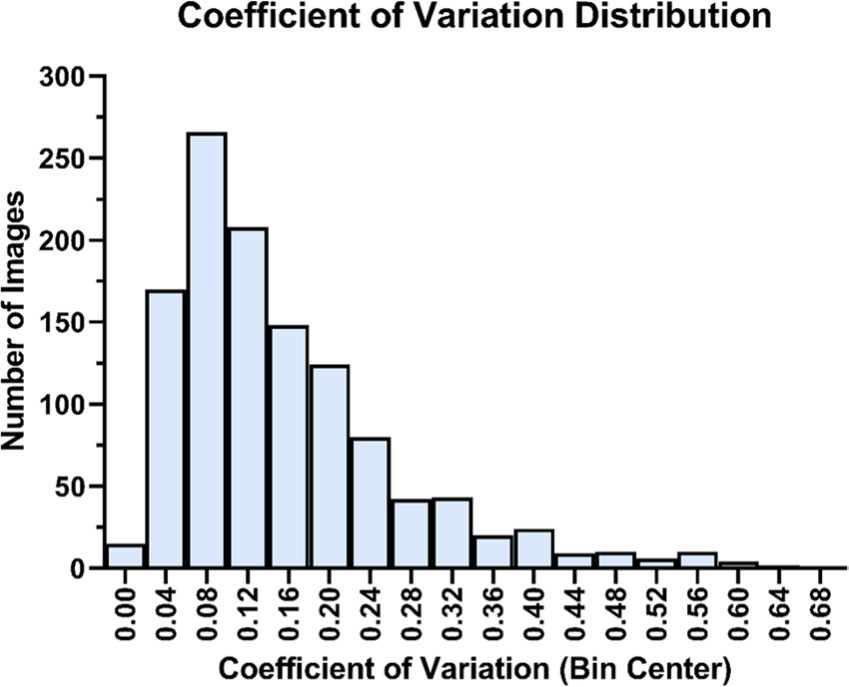
Histogram of Manual Count Variability for Rat Dataset. Variability between counters is expressed as the coefficient of variation (standard deviation of the manual count divided by the mean of the manual count for each image). The median coefficient of variation was 0.12, indicating good general agreement between manual counters.

Selected sub-images varied in image quality and contrast, and were from optic nerve sections that varied in tissue staining intensity and degree of nerve damage (Fig. 1). The images in our dataset can be viewed using the code found at github.com/ethier-lab/AxoNet. Four trained counters manually annotated “normal”-appearing axons in 1184 sub-images, where a “normal” axon was defined as a structure with an intact and continuous myelin sheath, a homogenous light interior, and absence of obvious swelling or shrinkage^5,8^. Each counter annotated one point per axon at the axon’s approximate center. The remaining 290 sub-images were annotated by two counters, who annotated one point per axon at the approximate center by consensus. Axons with abnormal morphology were not annotated. Counters were instructed to count axons which lay fully inside the frame of the image or which intersected either the left or top image border and lay more than halfway within the image borders. Manual annotations were made using Fiji’s Cell Counter plugin^23^, which recorded the spatial location of each axon marked within the image. There was good agreement between manual counts for most sub-images (Fig. 2).

These manual annotations were then used to create a “ground truth” axon count density matrix for each sub-image, ***D***, in which the (*i, j*)^th^ entry in the matrix was defined as

1 $$D(i,j)=\frac{1}{K}\mathop{\sum }\limits_{k=1}^{K}{c}_{k}(i,j),$$

where

$${c}_{k}(i,j)=\{\begin{array}{l}1,\,{\rm{if}}\,{\rm{the}}\,{(i,j)}^{{\rm{th}}}{\rm{pixel}}\,{\rm{was}}\,{\rm{annotated}}\,{\rm{by}}\,{\rm{the}}\,{{\rm{k}}}^{{\rm{th}}}\,{\rm{counter}}\\ 0,\,{\rm{otherwise}}\end{array}$$

and *K* was the number of counters for the sub-image in question. Note that the dimensions of ***D*** equaled the dimensions (in pixels) of the corresponding sub-image. Entries in ***D*** were then distributed (“blurred”) according to ${{\boldsymbol{D}}}_{{\boldsymbol{dist}}}={\mathscr{G}}({\boldsymbol{D}})$, where ${\mathscr{G}}$ is an isotropic Gaussian blur operator with $\sigma =8$ and filter size of 33 pixels, chosen empirically to distribute the annotated density values $D(i,j)$ over the full axon. This operation resulted in some of the annotated density values lying outside the edges of the original sub-image. This is a desired effect as an object which lies partially on an image’s boundary should not be counted as a full object^16^. The resulting ground truth matrix ***D***_***dist***_ provided the spatial distribution of axon count density over the full sub-image, which when summed over all entries, produced the ground truth axon count for the full sub-image or the average count from all experts for that sub-image. All density map values were stored as double-precision floating-point numbers.

#### Dataset subdivisions

The dataset was randomly divided into training, validation, and testing image subsets following a 60–20–20% split^24^. Images selected from each animal’s optic nerves were used exclusively for either the training, validation, or testing subsets. AxoNet was trained using the training subset. The validation subset was used to optimize AxoNet’s architecture and hyperparameters as well as to construct axon count correction equations, as was done using the calibration set in Reynaud *et al*.^12^ and as described in the Correction Equations section. Finally, the testing subset was used for final evaluation of tool performance.

### NHP dataset

We then evaluated the performance of AxoNet on optic nerve sub-images from NHPs with experimental glaucoma. This dataset had been previously annotated using a semi-automated manual method and used to develop one of the existing automated axon counting tools, AxonMaster, as described in Reynaud *et al*.^12^.

NHP dataset images were randomly divided into validation and testing subsets following a 50–50% split to match the even proportion of images in the validation and testing subsets of our rat dataset. Each subset contained 247 images. The validation subset was used to construct axon count correction equations, as was done using the calibration set in Reynaud *et al*.^12^ and as described in the Correction Equations section. The testing subset was used for final evaluation of performance for each tool.

### AxoNet development

#### Implementation and network architecture

We implemented a U-Net based encoder/decoder architecture similar to the original architecture developed by Ronnenberger *et al*.^15^. Specifically, we reduced the number of filters in our convolutional layers by a factor of two, resulting in a feature depth at each layer half of that in the original architecture. This reduction by a factor of two was chosen to reduce the number of parameters in the network, decreasing training time and reducing the danger of overfitting, while retaining the base-two relationship between the feature depths of the encoding and decoding paths of the U-Net. We also tried reducing the feature numbers by a factor of four, but this reduction decreased network performance. We used a rectified linear unit (ReLU) instead of a sigmoid activation for the final layer, indicated by the red arrow in Supplementary Fig. 1. The change in the final layer allowed us to regress the ground truth pixelwise count density function instead of predicting cell segmentation. A ReLU activation layer is better suited for this task because it produces a linear range of output pixelwise density map values, while a sigmoid activation biases its outputs towards either 0 or 1. We also included padding on all convolutional layers so that feature arrays would not shrink after each convolution. This network was implemented in Python (Version 3.7.3, Python Software Foundation) using Keras^25^ and Tensorflow^26^. The images were normalized by subtracting the mean pixel value for the entire image from the pixelwise values and dividing the resulting pixel values by twice the standard deviation of the image pixel values. This ensured that all pixels with intensities within ±2 standard deviations of the mean fell within the range [−1.0, +1.0]. Finally, outlier pixels were set to either −1.0 or 1.0.

The network was trained for 25 epochs with a batch size of 1 image per step and a learning rate of 10^−4^. Our modified architecture was developed iteratively by training on the training subset of the rat dataset and evaluating on the validation subset of the rat dataset. Validation performance was used to compare architectures until performance stopped improving.

#### Training

We used the Adam optimizer^27^ to minimize a mean squared error loss function evaluated between ground truth and predicted count density function estimates for each image as follows:

2 $$L(X,\beta )=\frac{1}{N}\mathop{\sum }\limits_{n=1}^{N}{[\hat{D}({X}_{n},\beta )-m{D}_{dist}({X}_{n})]}^{2},$$

where *β* is the learned network parameter set, *N* is the number of pixels in the image, $\hat{D}$ is the predicted pixelwise axon count density function, *X*_*n*_ is the n^th^ pixel in image *X*, and *m* is a density scaling factor. The density scaling factor was used to increase the magnitude of the predicted pixelwise density values, allowing better regression convergence. Its value was determined during hyperparameter optimization, resulting in a final value of *m* = 1000. Since a density scaling factor was used, the trained network overestimated the density predictions by a factor of *m*. Thus, all density maps predicted during network application were divided by *m* to accurately reflect ground truth. After density map prediction, we estimated total axon count within an image as follows:

3 $$Axon\,Count(X,\beta )=\frac{1}{m}\mathop{\sum }\limits_{n=1}^{N}\hat{D}({X}_{n},\beta \,).$$

Because dataset sub-images were randomly selected from larger full optic nerve images, their edges could contain cropping artifacts such as axons that intersected the edge. Dataset images and ground truth arrays were thus padded during training and evaluation through the edge-mirroring process recommended in^15^ to prevent the propagation of influence from these edge artifacts and any resulting biases in cell count. When computing the mean squared error loss function (Eq. 2), we did not include mirrored pixels. Training images were resized from 187 × 187 pixels to 192 × 192 pixels and extended to 224 × 224 pixels by this edge mirroring, as this size provided the optimum balance between training speed and output accuracy. Extensive data augmentation was used during training. This included image mirroring and rotation at intervals of 90° as well as random multiplicative pixel value scaling. The random multiplicative pixel value scaling was applied by taking the elementwise product of the training image matrix with a matrix of the same shape containing uniformly distributed values between 0.85 and 1.15.

As expected, our dataset contained only a few images with extreme numbers of axons per image, i.e. very low or very high axon counts. Training with this dataset would therefore lead to higher error in such cases, which we wished to avoid since having few axons per image or many axons per image can be a significant experimental outcome. If we denote the number of manually counted axons per image by manual counts (MC), then we reasoned that we could reduce counting error in extreme cases by creating a data set which had a more uniform distribution of MC over all the images, which we achieved by resampling, as follows. A 10 bin histogram of MC over all images in the training set was created, and we augmented the number of images in any bin that had less than the maximum number of images. This augmentation consisted of replicating all of the images within that bin until the number of images within each bin was approximately the same.

The model did not show signs of overfitting, as shown by the similar loss values for the training and validation set over the course of model training (Supplementary Fig. 2).

### Model Evaluation

#### Correction equations

The three automated counting tools, AxoNet, AxonMaster, and AxonJ, cannot precisely replicate ground truth. However, empirical observation shows that each tool demonstrated a relatively consistent bias, which could be corrected for. We therefore first used the validation subsets to perform the following linear bias correction, following the method established in Reynaud *et al*.^12^. In brief, MC and automated counts (AC) of axons in the validation subset were plotted against one another and fit using a linear least squares regression for each tool,

4 $$AC=a\,MC+b,$$

where coefficients *a* and *b* reflect any systematic linear bias in the estimation of MC by AC for the automated counting tool being considered. We then account for this linear bias by defining a corrected automated count, *AC*_*corrected*_, as

5 $$A{C}_{corrected}=\frac{AC-b}{a}.$$

Ideally, our automated counting methods would not demonstrate any systematic bias, i.e. our network would learn to correct any such biases during training. However, all automated counting schemes that we are aware of either show some bias, and thus use linear bias correction equations, or do not report results in a manner that allows one to determine whether the scheme shows bias, e.g. by reporting only mean absolute error between ground truth and object counts. We have chosen to use correction equations.

We conducted a series of tests to determine the cause of this systematic linear bias. Specifically, we first intentionally overfit models to determine whether there was a bug in our code causing systematic bias, reasoning that if we could eliminate bias by overfitting, then bias would not be due to a programming error and instead would be related to other factors. We thus trained on downsampled training sets with and without resampling and augmentation and evaluated the bias when the algorithm was applied to the same downsampled training sets. Second, we tested for underfitting by training our network with randomized initial parameters and for more epochs and comparing the results to our initial results. We also considered differences between the training and testing sets and dataset imbalance as potential sources of bias.

#### Statistical analysis of tool performance on the rat image dataset

To evaluate the three automated counting tools on the rat image dataset, we applied all three tools to the validation subsets, created correction equations as described above in Eq. (5), and applied the relevant correction equation to the automated counting results from the testing subset. Differences in sub-image manual counts and the automated counts produced by each automated axon counting tool were quantified for both datasets through linear regressions, Kruskal-Wallis tests comparing the mean absolute error for each tool, and a comparison of the limits of agreement as defined by the Bland-Altman methodology^28^.

In more detail, after linear regression between manual and automated counts, we examined the residual distributions from the regressions, and discovered they were not normally distributed (Shapiro-Wilk test, all p < 0.05). However, inspection of the data by histogram and Q-Q plot showed approximate normality with the exception of a small number of outliers and a slight heteroscedasticity for each distribution. In addition, linear regression is known to be robust to such slight deviations from normality, particularly in larger data sets like ours^29,30^. We therefore judged these deviations from normality to be minor, and continued to use simple linear regression to compare model performance, taking a larger R^2^ value to indicate a more consistent agreement between manual and automated counts.

We also calculated the mean absolute error between each automated counting tool’s axon count and the gold-standard manual axon counts to quantify the accuracy for that tool. None of the mean absolute error distributions for each tool’s results were normally distributed (Shapiro-Wilk: all p < 0.001), so we compared the tools’ mean absolute errors using the Kruskal-Wallis test with Dunn’s post hoc test.

Finally, we used Bland-Altman plots^28^ to compare the limits of agreement calculated for each method. Ideally, the errors from the automated tools would lie within the range of inter-observer variability. Thus, we aimed for the limits of agreement of these Bland-Altman plots (mean count error ± 1.96•SD of count error) to be within the limits of agreement calculated for individual counters’ MC relative to the mean MC. Using this definition, we computed the limits of agreement for our rat dataset as ±14.3 axons. Additionally, for each image with four manual counters (1184 of 1514 images), corrected ACs were compared to a 95% confidence interval constructed from the four MCs. We defined a success rate as the proportion of images for which the corrected AC fell within this 95% confidence interval. This approach evaluated both automated counting accuracy and precision in the same measurement.

#### statistical analysis of tool performance on the nhp image dataset

We also evaluated our rat-trained AxoNet algorithm and the two existing axon counting tools on the NHP dataset. To do so, we applied all three tools to the validation subset, created correction equations as stated above, and then applied the correction equations to the automated counting results from the testing subset. Relationships between semi-automated manual (SAM) and corrected automated counts were assessed in the same manner as they were in the rat image dataset. Since only mean axon counts were available, we were unable to compute the proportion of the automated counts that fell within the 95% confidence interval for the SAM counts or define a desired range for the limits of agreement as we did for the rat optic nerve image dataset. However, we were able to compare the limits of agreement between the corrected ACs and the SAM counts.

## Results

### Rat model dataset results

**Figure 3:**
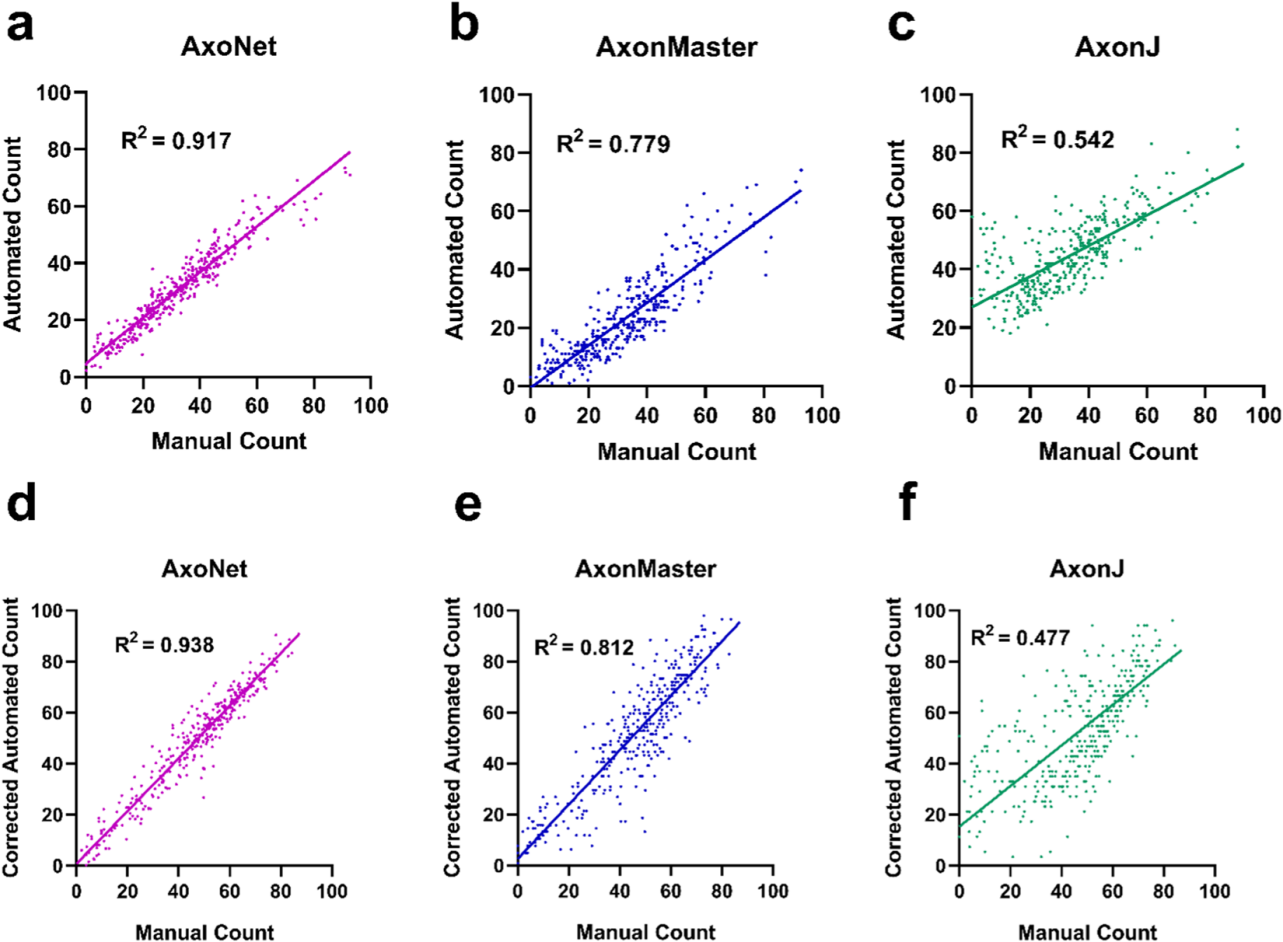
Comparison between automated and manual axon counts for the rat validation and testing subsets. Validation subset results are shown for AxoNet (**a**), AxonMaster (**b**) and AxonJ (**c**). The regression relationships between MC and AC counts were: AxoNet: AC = 0.801*(MC) + 4.8; AxonMaster: AC = 0.731*(MC) − 0.633; and AxonJ AC = 0.508*(MC) + 26.2. These relationships were used as correction equations when counting axons in the testing subset. Testing subset results are shown for AxoNet (**d**), AxonMaster (**e**) and AxonJ (**f**). Testing subset mean absolute errors are 4.4, 12.8, and 9.5 axons for AxoNet, AxonMaster, and AxonJ respectively. AC values are shown after applying the correction equations from the validation subset results. Each data point is obtained from a single sub-image from the corresponding subset.

We first applied the three automated counting tools to the validation subset of the rat dataset to determine correction equations that accounted for linear bias, as described above (Fig. 3). We then applied the automated tools to the testing subset. Before compensating for linear bias using the correction equations, the relationship between AxoNet automated and manual counts (AC and MC) in the testing subset was AC = 0.826*(MC) + 5.36 (R^2^ = 0.938), indicating a comparable bias to that seen when our model was applied to the validation subset. For all three automated tools, the corrected linear fit between *MC* and *AC*_corrected_ resulted in regression slopes and intercepts that were mostly significantly different from 1 and 0, respectively (t-test for slope, all p < 0.05; t-test for intercept, p = 0.0319, p = 0.059, p < 0.001; all p-values presented in the order: AxoNet, AxonMaster, and AxonJ; Fig. 3). These findings indicate that the correction equation method did not fully correct for consistent linear biases, although their effects were reduced.

**Figure 4:**
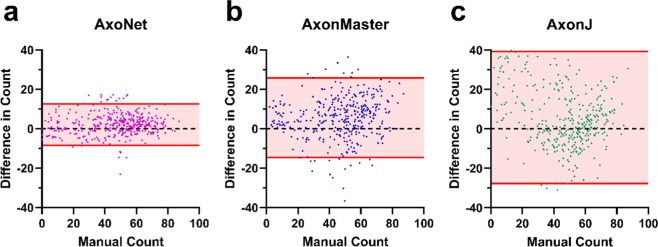
Comparison of error distribution for the rat testing subset. Differences between rat testing subset MC and corrected AC are plotted against manual counts for AxoNet (**a**), AxonMaster (**b**) and AxonJ (**c**) as Bland-Altman plots. Each data point is a single sub-image from the rat testing dataset. Red lines represent the upper and lower bounds for the limits of agreement, calculated as mean error ± 1.96*(standard deviation of error). Limits of agreement are [−8.3, 12.6], [−14.59, 25.8], and [−27.7, 39.4] axons for AxoNet, AxonMaster, and AxonJ, respectively.

Of the three tools, AxoNet achieved the highest correlation between its corrected AC and the MC (R^2^ = 0.938) as well as the smallest mean absolute error (Kruskal-Wallis: Chi-square = 169.7 and p < 0.001; Dunn’s post-hoc: all p < 0.001, Fig. 3). Only AxoNet demonstrated limits of agreement within the threshold determined by the manual count agreement (Fig. 4). For the images annotated by four counters, the percentage of corrected ACs that fell within the 95% confidence interval of the manual counts was 83%, 48%, and 58% for AxoNet, AxonMaster, and AxonJ respectively. Taken together, we observe that AxoNet performed the best (i.e. the closest to manual annotations) on the testing subset of the rat dataset.

**Figure 5:**
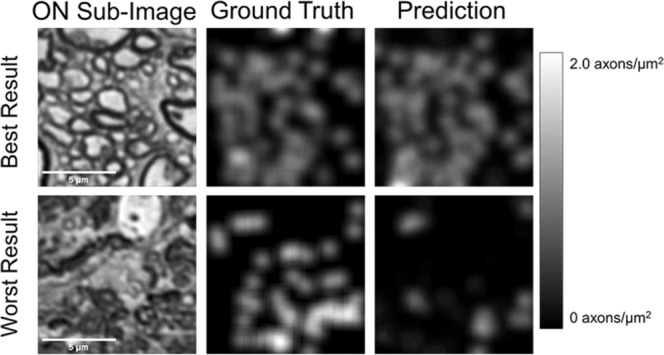
Visualization of AxoNet Performance. The images from the rat testing subset which produced the smallest (top) and greatest (bottom) difference between AxoNet predicted and ground truth manual axon count are shown in the left column. The corresponding manually annotated ground truth axon count density maps are shown in the middle column, and the automatically detected axon count density maps are shown in the right column. The scale bar on the right shows the map used to visualize axon count density as greyscale intensity.

We also visualized the output of AxoNet by determining whether AxoNet was accurately replicating the density maps used during its training by comparing its predicted spatial axon count densities to ground truth (Fig. 5). Generally, the density maps produced by AxoNet matched those produced by the manual annotators.

### NHP dataset results

**Figure 6:**
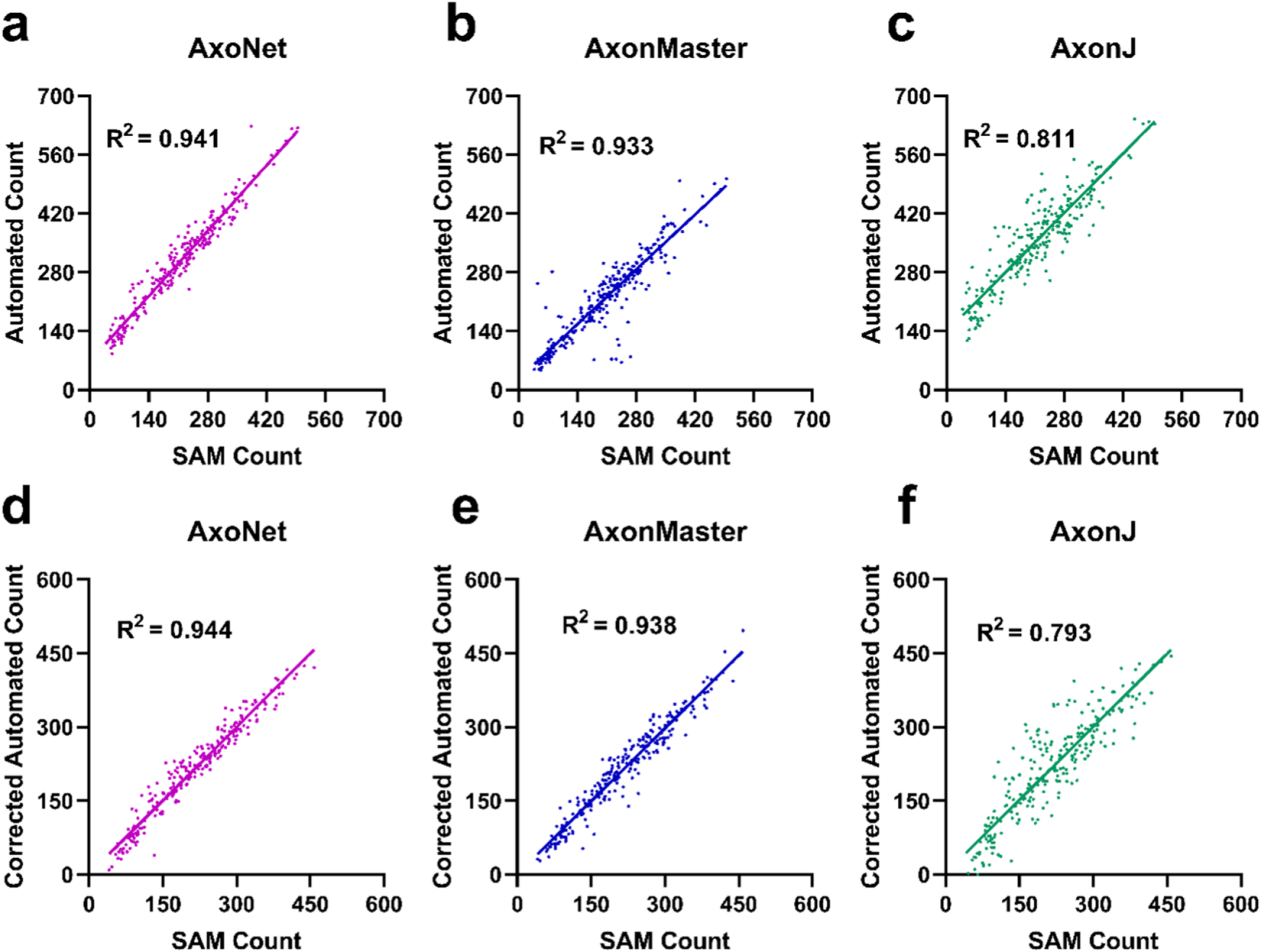
Comparison between automated and manual axon counts for the NHP validation and testing subsets. Validation subset results are shown for AxoNet (**a**), AxonMaster (**b**) and AxonJ (**c**). The regression relationships between SAM and AC counts were: AxoNet: AC = 1.11*(SAM) + 69.0; AxonMaster: AC = 0.9849*(SAM) + 17.4; and AxonJ AC = 1.01*(SAM) + 139.2. These relationships were used as correction equations when counting axons in the testing subset. Testing subset results are shown for AxoNet (**d**), AxonMaster (**e**) and AxonJ (**f**). Testing subset mean absolute errors are 17.7, 18.2, and 35.0 axons for AxoNet, AxonMaster, and AxonJ respectively. AC values are shown after applying the correction equations from the validation subset results. Each data point is obtained from a single sub-image from the corresponding subset.

We then applied these three automated counting tools to the NHP dataset. We first assessed the performance of the three tools using the validation subset of the NHP dataset in order to construct bias correction equations relating each tool’s AC to the SAM count. When applied to the validation subset of the NHP dataset, AxoNet achieved a higher correlation between SAM count and AC than the other two tools, although AxonMaster needed less bias correction (Fig. 6), likely because it had been optimized for the NHP dataset.

**Figure 7:**
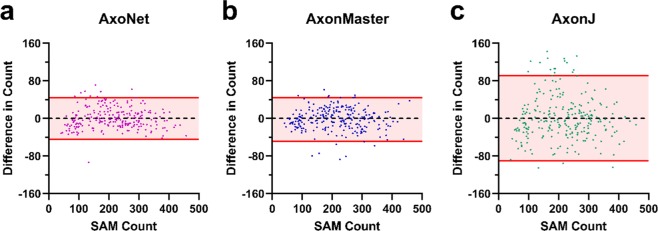
Comparison of error distribution for the NHP testing subset. Differences between NHP testing subset semi-automated manual count *and corrected AC are plotted against* semi-automated manual count for AxoNet (**a**), AxonMaster (**b**) and AxonJ (**c**) as Bland-Altman plots. Each data point is a single sub-image from the rat testing subset. Red lines represent the upper and lower bounds for the limits of agreement, calculated as mean error ± 1.96*(standard deviation of error). Limits of agreement are [−43.9, 42.8], [−48.9, 47.5], and [−91.0, 93.4] axons for AxoNet, AxonMaster, and AxonJ respectively.

The automated counting methods and their correction equations were then applied to the testing subset of the NHP dataset to directly compare their ability to accurately quantify the number of axons present in each image. For all three automated tools, the corrected linear fit between SAM count and *AC*_corrected_ resulted in regression slopes and intercepts that were not significantly different from 1 and 0, respectively (t-test for slope, p = 0.77, p = 0.47, p = 0.81; t-test for intercept, p = 0.77, p = 0.82, p = 0.71; all p-values presented in order: AxoNet, AxonMaster, and AxonJ; Fig. 6). Of the three tools, AxoNet achieved the highest correlation between its corrected automated and manual counts (R^2^ = 0.944), with AxonMaster achieving a comparable correlation (R^2^ = 0.938). AxoNet and AxonMaster both had lower mean absolute error when compared to AxonJ (Kruskal-Wallis: Chi-square = 62.57 and p < 0.001; Dunn’s post-hoc: both p < 0.001, Fig. 6), while AxoNet and AxonMaster had similar mean absolute error values to one another (p > 0.9). AxoNet and AxonMaster produced comparable limits of agreement, whereas AxonJ’s limits of agreement were larger (Fig. 7).

**Figure 8:**
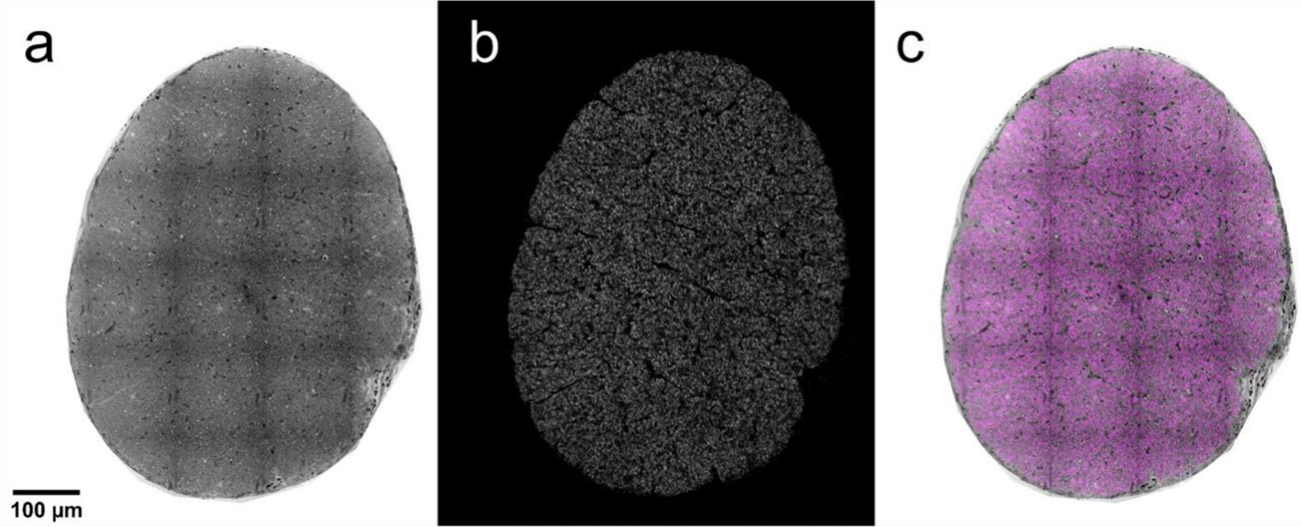
AxoNet Plugin Results. After using the AxoNet plugin for ImageJ and Fiji on an image of a full rat optic nerve (**a**), the output axon density map (**b**) and the combination of these two images (**c**) are displayed. The combination of these two images is shown with the input image (**a**) in greyscale and the axon density map (**b**) overlaid in pink. Axon density scale is not provided here because these full images are scaled down significantly for inclusion in the manuscript and color scale is indistinguishable at this resolution. A grid of dark lines is visible in panel a; these lines correspond to tile edges from the microscopy imaging and are an artifact of visualization only since counts are carried out on much smaller portions of the full image.

We packaged AxoNet into a user-friendly plugin for Fiji and ImageJ. This plugin is capable of counting full rat optic nerve images in about 15 minutes (Fig. 8). We typically count c. 80,000 “normal”-appearing axons in a healthy nerve, consistent with previous reports^5,6,31^.

### Bias

To investigate the source of the small bias seen in AxoNet, i.e. the fact that there was a difference between the unity line and the best fit regression lines in Figs. 3 and 6, we conducted several experiments. In constructing these experiments, we considered the following possible sources of error: (1) a bug in the algorithm; (2) poor convergence of our parameter values during the training phase, i.e. underfitting; (3) inherent differences between the training and testing data sets; and (4) tendency of the algorithm to be biased towards the majority group, which in our case was images with axon counts close to the dataset mean axon count^32^. We consider each of these in turn.

1. *Bug in the algorithm:* We conducted experiments in which we intentionally overfit the network, as follows. We first trained AxoNet on subsets of the full training set of different sizes, and then evaluated the algorithm on those same images. Within this framework, training was conducted using three variations of the training image sets: images that were neither augmented nor resampled, images that were augmented but not resampled, and images that were resampled but not augmented. As data set size decreased, bias decreased; indeed, training on a single repeated image and testing on the same image produced essentially zero error (less than one axon; Supplementary Fig. 3). Because we were able to essentially eliminate bias by overfitting, we concluded that a bug in the code used to train or assess our network was unlikely.
2. *Underfitting*. Our numerical experiments suggested that the parameter optimization process had converged. Specifically, we found that increasing the number of epochs during training did not improve convergence, as measured by the loss function’s final value. Further, using different initial parameter values yielded essentially the same loss function values at the end of training. Thus, we do not believe that bias was due to underfitting.
3. *Inherent differences between data sets:* Error can arise if the training, validation and testing data sets have systematic differences. Such an error source is inherent in supervised machine learning approaches^33^. In our case, we saw that the bias differed between validation and testing data sets (compare Figs. 3 and 6), suggesting subtle systematic differences between image sets. Consistent with this suggestion, the bias was reduced if we trained and tested on the same data set, while image augmentation increased bias if testing was conducted on the training data set (Supplementary Fig. 3; compare red with black symbols). It is interesting to note that training set resampling slightly increased the mean absolute error (compare red with green symbols in Supplementary Fig. 3). We suggest that this occurs because resampling increases the proportion of “hard to count” images, i.e. those with extensive damage or many small axons. However, resampling also reduces error when evaluating different testing and training data sets, and thus is still recommended.
4. *Bias towards the mean:* We note that the algorithm consistently overcounted images with small numbers of axons, and undercounted images with large numbers of axons, suggesting bias towards the mean. Research on evaluating the effects of class imbalances on neural network training for classification and regression shows a similar effect^32,34,35^, where in our case, images with counts closer to the dataset mean are analogous to the “majority class”. To reduce the magnitude of this effect, we resampled our training set images to produce a uniform distribution of axon counts (see Methods). Even when doing so, a small systematic bias remained, which perhaps reflects a tendency of bias towards the mean even when training occurs on a uniformly sampled image set. Nonetheless, this bias was small and considered acceptable in this application.

## Discussion

The purpose of this study was to develop and evaluate a new approach to automatically count “normal”-appearing RGC axons in a diverse dataset of healthy and damaged optic nerve cross sections. Such an automated axon counting tool is a useful tool in studying glaucoma and potentially other neurodegenerative disorders. We designed this new approach to work well over a range of image qualities and for multiple mammalian species. AxoNet’s predicted axon counts proved to be highly correlated to manual axon counts in both the rat and NHP datasets, indicating that it met our requirements for an automated axon counting tool. As judged by the uniform error over the range of manual axon counts (Figs. 4 and 7), AxoNet performed equally well on images of damaged vs. healthy optic nerves. This is significant because axon counting is more difficult in diseased tissue, and suggests promise for the use of AxoNet as a tool for nerve damage analysis in experimental glaucoma.

Prior to building AxoNet, we explored the methodologies previously used to create existing automated axon counting tools. AxonMaster uses a fuzzy c-means classifier as an adaptive thresholding method to segment axon interiors from the darker myelin sheath. These clusters are then filtered by size and circularity before counting axons. AxonJ uses a Hessian operator to identify the darker myelin sheath and then performs similar adaptive thresholding and connected region size filtering region before counting the connected regions as axons. When applied to the rat dataset, these two tools produced adequate segmentation of total axon area in optic nerve images, but often did not produce accurate segmentation of individual axons, leading to inaccurate counts. We also attempted to apply two other segmentation techniques, ilastik^36^ and the basic pixel segmentation U-Net^15^. These approaches also resulted in inaccurate counts, especially when applied to damaged tissue; therefore, we adapted an alternate cell counting framework introduced by Lempitsky *et al*.^16^. This approach avoids the difficult task of semantic segmentation and instead predicts a pixelwise cell count density estimate. The authors accomplished this through using machine learning with hand-crafted pixelwise features^16^. More recently, attempts have been made to perform similar count density function estimations using convolutional neural networks^37^ and adapted U-Net architectures^38^ for crowd counting, which is a technically similar problem to cell counting. Convolutional neural networks have also been used recently for axon segmentation in scanning and transmission electron microscopy images of mammal and human spinal cord^39^. The tool produced in this work is the result of this synthesis between a convolutional neural network architecture designed for cell segmentation, the U-Net, and a count density prediction strategy. This method avoids the hard problem of axon segmentation in lower-resolution light microscopy, trading the ability to analyze single-axon morphology for the most accurate axon count.

This study was limited by several factors. First, and most important, to date AxoNet has been trained to count only “normal”-appearing axons, similar to existing axon-counting software. The classification of an axon as “abnormal” in appearance does not necessarily imply that the axon is non-functional, and thus our tool may not count axons that are in fact conducting visual information. However, due to AxoNet’s generalizability and lack of reliance on hand-crafted features specific to “normal”-appearing axons, it can be extended to count or even segment other features of both healthy and glaucomatous optic nerves, such as glial processes, nuclei, “abnormal” axons, large vacuoles, and extracellular matrix. We are currently extending AxoNet to quantify these features. Such analysis of features beyond “normal”-appearing axons may provide new insight into the pathophysiological processes of glaucomatous nerve degeneration.

Second, we were unable to fully eliminate systematic biases in the network’s predictions. We investigated the source of this systematic prediction bias and found that it did not originate from errors within the training or prediction code or from underfitting. We posit that some bias may be unavoidable due to subtle differences in the training and testing sets, which can be mitigated by increasing the variability within the training data set. Based on the literature, we also posit that training set imbalance may cause training bias towards common training cases. This source of bias can be mitigated by resampling rarer cases to increase their influence during network training. By doing so, we were able to reduce bias to only a few axons per image. Considering the complexity of our images and the variability from animal to animal in glaucoma models, we judged this level of bias to be acceptable.

Third, the linear bias correction equations determined in this study were suitable for countering systematic bias in our data set, but may not necessarily be accurate for other data sets, since the conditions which create these systematic biases may vary with experimental treatment, imaging protocols, or tissue processing protocols. However, we do not expect such effects to be severe, since we intentionally included these sources of variability within the two image datasets used in this study and AxoNet still performed well. Nonetheless, it would be prudent to calibrate AxoNet for each new application, which can be done through using correction equations like those created with our validation subsets or network retraining with a new dataset according to the training protocol detailed above.

A fourth limitation is that all manual counts were conducted by members of one lab, and it is possible that manual counts generated in different research groups could be slightly different from ours since manual counting itself is not entirely unambiguous. This uncertainty is inherent in axon quantification and cannot be avoided, although to enhance repeatability we have explicitly described our definition of “normal”-appearing axons and have made the training data publicly available.

Presently, AxoNet regresses a pixelwise count density function which is integrated over the full image to return a count. Fitting the density function is accomplished through the minimization of a mean squared error loss function evaluated at each pixel (Eq. 2). This loss function may be overly sensitive to zero-mean noise and other variations in training images. Lempitsky *et al*.^16^ originally solved this problem through the Mesa loss function, which used a maximum subarray algorithm to find the image region with the largest difference between automated and manual counts and minimized count error over this region instead of at every pixel^16^. When we attempted to use this loss function during our training, the resulting method was far too computationally expensive and resulted in a prohibitively long training time (on the order of hours per training step). However, developing a new loss function which avoids computing the mean square error at every pixel per iteration but does so without the computational expense may improve AxoNet’s performance in terms of accurate axon count insensitive to image noise.

The successful use of the rat-trained AxoNet to count NHP images is indicative of the versatility of our method, even without re-training. However, the network can be easily re-trained on a new counting case if needed. If there is adequate training data in the new set, the deep learning framework can adapt itself to new applications without requiring any changes in handcrafted features. Data augmentations like those described in the methods can be applied to improve network learning from limited datasets, as was done in the first published application of the U-net architecture^15^.

We can also use AxoNet to count axons in full rat optic nerve images by subdividing the full image into tiles for individual processing. This tile-based processing was necessary because of the prohibitive computational expense involved in applying the U-net architecture to large images. However, tile-based processing has the potential to create edge artifacts by cutting off portions of cells on the borders of each tile. We correct for this potential error by padding the edges of each processing tile with bordering pixels from adjacent processing tiles. Including this information from bordering tiles meant that the resulting density map prediction was not affected by these potential tile cropping artifacts. Once processed, the resulting density map was cropped back to its original tile size. This padding was not done when it would have required pixels from beyond the image boundaries. These padded tiles were then also mirrored, as described for model training above.

When running on the system used for this study (Windows Desktop, Intel i7–3770 CPU at 3.40 GHz, 16 GB RAM; Dell, Round Rock, TX), AxoNet counts the axons within a full rat optic nerve image in approximately 15 minutes. For comparison, it took AxonJ and AxonMaster approximately 30 minutes and 1 hour, respectively, to count the axons within a full rat optic nerve image. Therefore, our tool can be applied to analyze full optic nerve images with runtimes comparable to, or better than, those of the existing automated tools.

## Conclusion

We have successfully applied a deep learning method to accurately count “normal” axons in both rat and non-human primates, and in both healthy and experimentally glaucomatous optic nerve sections. Additionally, we have compared AxoNet to two previously published automated counting tools and shown that AxoNet performs as well as or better than these two tools in counting healthy axons in these two datasets. Our tool is available online as an ImageJ plugin and can be installed by following the instructions at https://github.com/ethier-lab/AxoNet-fiji. The code and data we used to train the model can be found at https://github.com/ethier-lab/AxoNet.

## Supplementary information

Supplementary Information.

## Data Availability

The rat optic nerve image dataset generated and analyzed during the current study are available in the Github repository, https://github.com/ethier-lab/AxoNet. A spreadsheet containing the count data for each image in both the NHP and rat datasets is available on the same repository. The NHP optic nerve image dataset and AxonMaster software are the property of the Burgoyne Lab, where they are available upon reasonable request.
