# Supplementary material for: AxoNet: A deep learning-based tool to count retinal ganglion cell axons
Source: Sci Rep. 2020 May 15;10:8034. doi: 10.1038/s41598-020-64898-1 (PMC7228952; doi:10.1038/s41598-020-64898-1)
Supplement: Supplementary file 1 — Supplementary Information. [file 41598_2020_64898_MOESM1_ESM.pdf]

AxoNet: A deep learning-based tool to count retinal ganglion cell axons

Supplementary Information

Matthew D Ritch[1], Bailey G Hannon[2], A Thomas Read[1], Andrew J Feola[1,3], Grant A Cull[4], Juan Reynaud[4], John C Morrison[5], Claude F Burgoyne[4], Machelle T Pardue[1,3], C Ross Ethier[1,2]*

1. Wallace H. Coulter Department of Biomedical Engineering, Georgia Institute of Technology and Emory University, Atlanta, Georgia, United States
2. George W. Woodruff School of Mechanical Engineering, Georgia Institute of Technology, Atlanta, Georgia, United States
3. Center for Visual and Neurocognitive Rehabilitation, Atlanta VA Healthcare System, Atlanta, Georgia, United States
4. Devers Eye Institute, Legacy Research Institute, Portland, Oregon, United States
5. Casey Eye Institute, Oregon Health & Science University, Portland, Oregon, United States

*Corresponding author:

Prof. C. Ross Ethier
ross.ethier@bme.gatech.edu
Georgia Institute of Technology and Emory University
Atlanta, GA

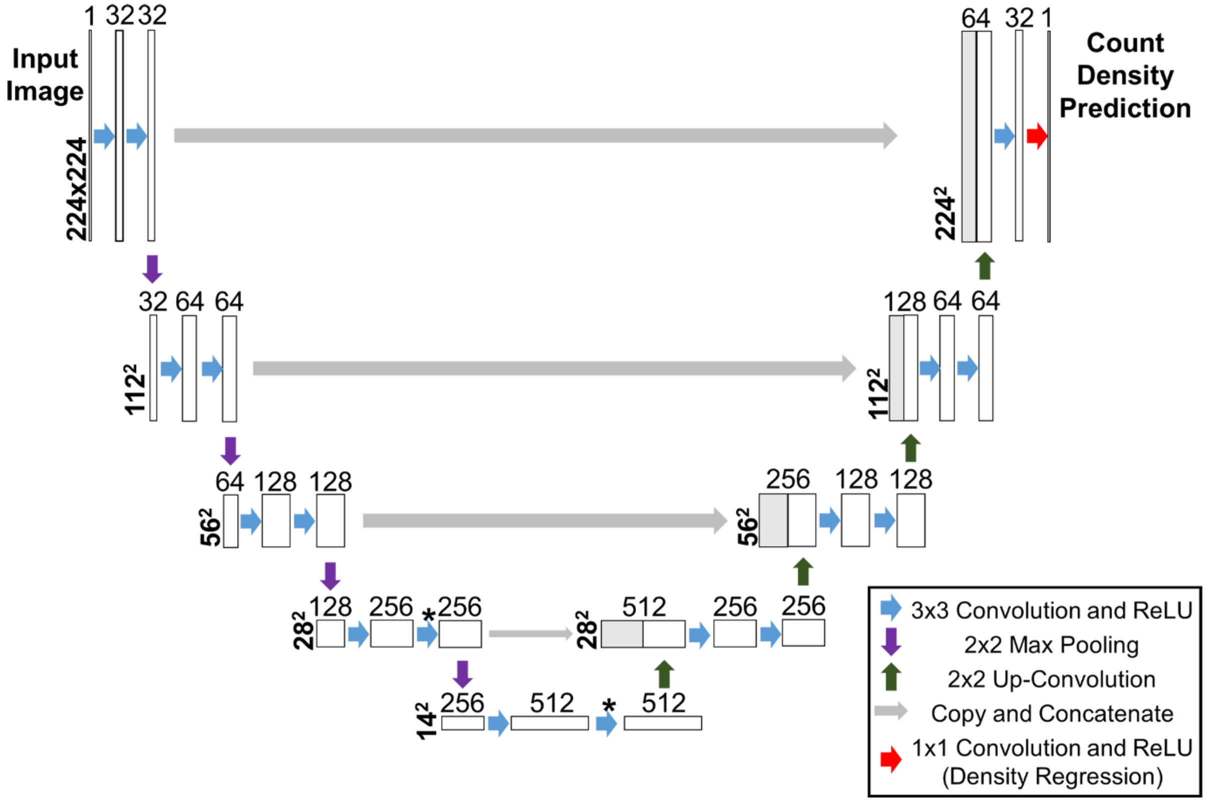

Supplementary Figure 1: U-Net Architecture. A visual representation of our adapted U-Net convolutional neural network architecture, with the encoding branch on the left and the decoding branch on the right. Each box represents the output array of one of the convolutional network's layer operations, which are represented by colored arrows. The bold numbers to the left of the boxes indicate the row and column sizes of the feature array at those layers. The numbers above the boxes indicate the feature depth of each layer, which is the third dimension of the feature array at that layer. Numbers in the layer operations key indicate the size of that operation's sliding window. Products of feature concatenation are indicated by two boxes sharing a border with the concatenated box in grey. The asterisk indicates dropout with rate = 0.5 applied after convolution. ReLU is an abbreviation for Rectified Linear Unit. Figure adapted from Ronnenberger et al. [15].

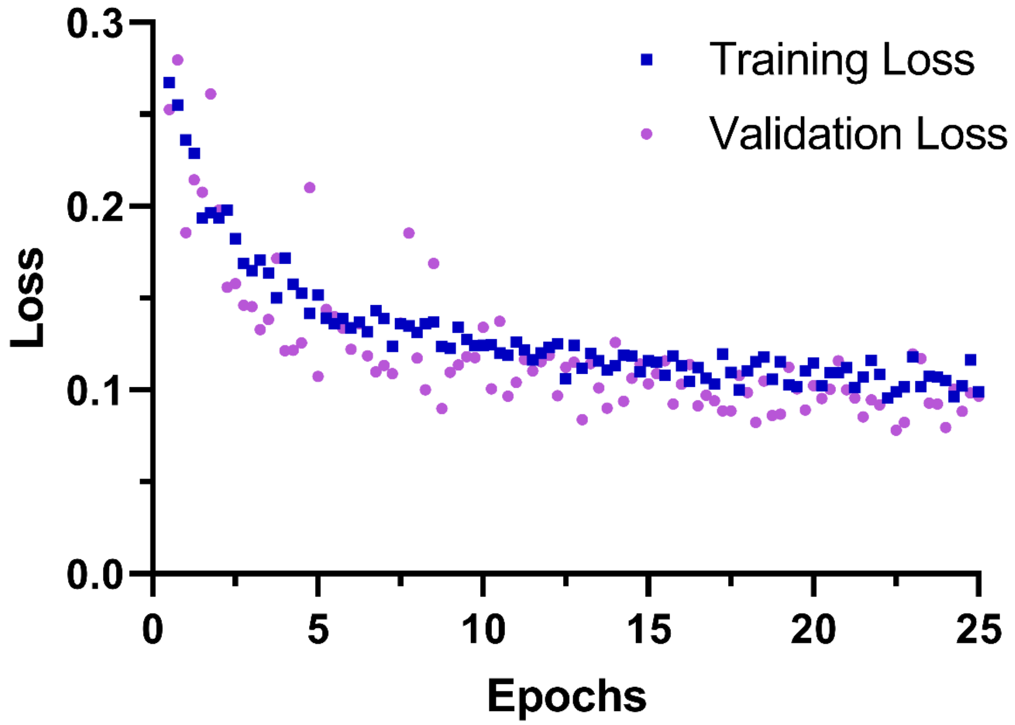

Supplementary Figure 2: AxoNet Training Loss. Average training and validation set loss for each epoch vs. epoch number. Training and validation set losses do not diverge, indicating that our network did not overfit during training.

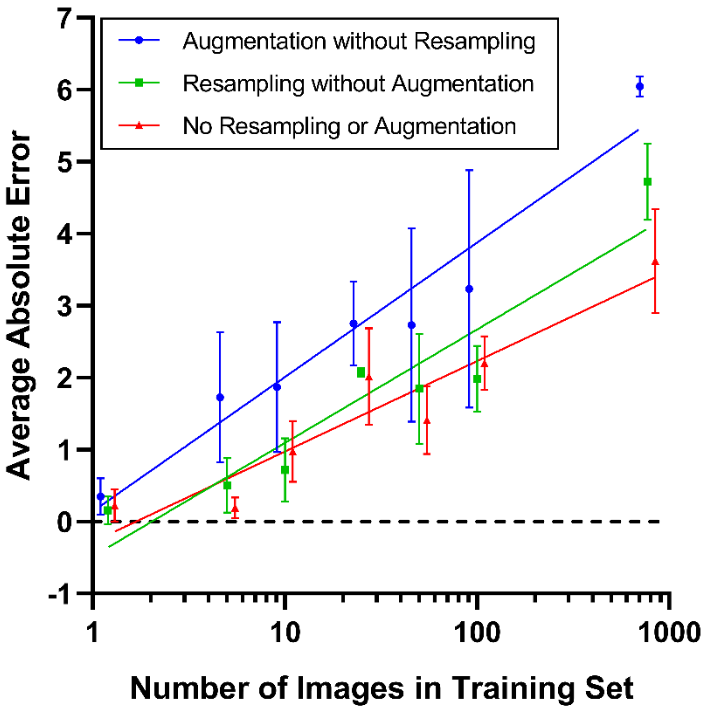

Supplementary Figure 3: Training Set Subsampling. The full training data set was sub-sampled to produce training data sets of different sizes (N). AxoNet was then trained with these sub-sampled sets, and subsequently used to count axons in the same data set, i.e. for purposes of generating this figure, the testing and training data set were the same for each realization. We computed a mean absolute error (MAE) over the data set and repeated this process 3 times for each training set size (i.e. 3 replicates) to obtain a mean and standard deviation of the MAE. This full analysis was completed for three training set conditions: training augmentation without resampling (blue symbols), resampling without training augmentation (green symbols), and no training augmentation or resampling (red symbols). The respective fitted relationships for these three conditions were MAE = 0.1 + 1.8*log(N), MAE = -0.4 + 1.5*log(N), and MAE = -0.2 + 1.2*log(N). Note that the horizontal axis is logarithmic and that plotted values at the same training set size are offset slightly in the horizontal direction for visual clarity.
